# Bacterial Biofilm Formation on Biomaterials and Approaches to Its Treatment and Prevention

**DOI:** 10.3390/ijms241411680

**Published:** 2023-07-20

**Authors:** Panxin Li, Rui Yin, Juanli Cheng, Jinshui Lin

**Affiliations:** Shaanxi Key Laboratory of Chinese Jujube, College of Life Sciences, Yan’an University, Yan’an 716000, China; 17729316249@163.com (P.L.); 17809114132@163.com (R.Y.); chengjl-1981@163.com (J.C.)

**Keywords:** biofilm formation, biomaterials, antibiotic resistance mechanisms, biofilm infection, antibiofilm strategies

## Abstract

Bacterial biofilms can cause widespread infection. In addition to causing urinary tract infections and pulmonary infections in patients with cystic fibrosis, biofilms can help microorganisms adhere to the surfaces of various medical devices, causing biofilm-associated infections on the surfaces of biomaterials such as venous ducts, joint prostheses, mechanical heart valves, and catheters. Biofilms provide a protective barrier for bacteria and provide resistance to antimicrobial agents, which increases the morbidity and mortality of patients. This review summarizes biofilm formation processes and resistance mechanisms, as well as the main features of clinically persistent infections caused by biofilms. Considering the various infections caused by clinical medical devices, we introduce two main methods to prevent and treat biomaterial-related biofilm infection: antibacterial coatings and the surface modification of biomaterials. Antibacterial coatings depend on the covalent immobilization of antimicrobial agents on the coating surface and drug release to prevent and combat infection, while the surface modification of biomaterials affects the adhesion behavior of cells on the surfaces of implants and the subsequent biofilm formation process by altering the physical and chemical properties of the implant material surface. The advantages of each strategy in terms of their antibacterial effect, biocompatibility, limitations, and application prospects are analyzed, providing ideas and research directions for the development of novel biofilm infection strategies related to therapeutic materials.

## 1. Introduction

A biofilm is a population structure comprising a highly structured complex formed by bacteria attached to various solid surfaces and their production of extracellular polysaccharides (EPSs), matrix proteins, and extracellular DNA (eDNA) [1]. Biofilms provide many biological advantages for bacteria, such as high infectivity, drug resistance, and strong viability [2]. It is estimated that more than 65% of nosocomial infections, about 80% of chronic infections, and 60% of human bacterial infections are caused by biofilms [3], including chronic biofilm diseases such as cystic fibrosis and periodontitis, as well as biofilm infections related to biomedical materials, such as medical device infections, implant-related infections, and others [4].

Biomaterial-associated infections (BAI) are one of the most common and feared complications in medical practice [5], typically stemming from the attachment of microorganisms to the surfaces of medical devices. These infections are mostly caused by Staphylococci, particularly *Staphylococcus epidermidis* and *Staphylococcus aureus*, as well as by Streptococci, Gram-negative bacilli, enterococci, and anaerobes, such as *Propionibacterium acnes* [6,7]. The biofilms formed by pathogens provide them with protection from therapeutic interventions, resulting in persistent and chronic infection [8]. As the population ages, there is an increasing need to restore and maintain the operability and quality of biomedical devices, and combined with the continuous improvement of technology in the medical field, the problem of BAI is expected to increase [9]. It has been reported that device-associated infections, including ventilator-associated pneumonia, catheter-associated urinary tract infections, and central catheter-related bloodstream infections, account for approximately 26% of all healthcare-associated infections in the United States [9]. The economic burden of treating device-associated infections is also extraordinarily high; in the United States, the average revision costs of infectious hip and knee arthroplasty are approximately USD 80,000 and USD 60,000, respectively [10]. It is worth noting that once a biofilm is formed on medical equipment, conventional methods often cannot effectively eradicate the biofilm and long-term antibacterial treatment is usually required [11]. As current antimicrobial therapy is expensive and will increase the drug resistance of pathogens, there is interest in the development of new antimicrobial therapies [12].

In this review, we introduce in detail the formation process and drug resistance mechanism of biomaterial-related biofilms and summarize their characteristics in clinical infections, such as persistent infections and inflammation. Two methods are discussed for the prevention and treatment of biofilm infection related to biomaterials, namely, antibacterial coatings against biofilm infection and the surface modification of biomaterials, which provides ideas for the development of new anti-biofilm treatments.

## 2. Biofilm Formation on Biomaterials

**Figure 1:**
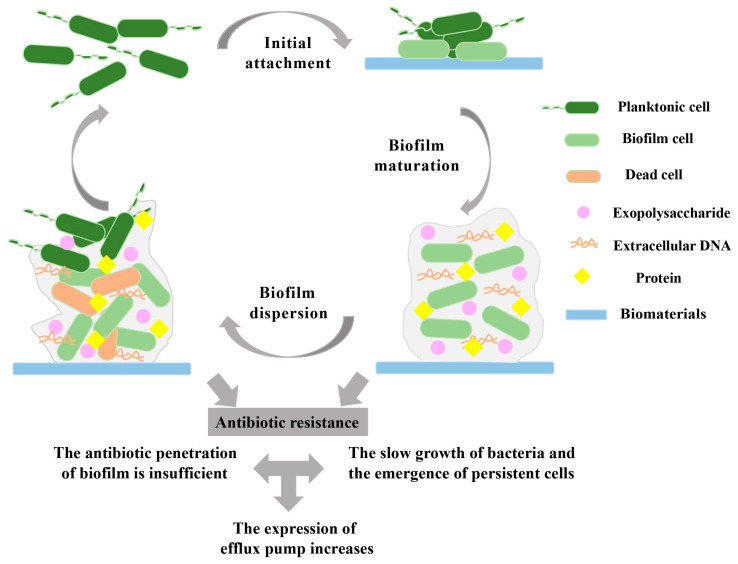
The process of biofilm formation and antibiotic resistance mechanisms. Biofilm formation is divided into three steps: Initial attachment of the biofilm; Biofilm maturation; Biofilm dispersion. The mechanisms of antibiotic resistance in biofilms are also divided into three factors: The antibiotic penetration of the biofilm is insufficient; The bacteria grow slowly, and persistent cells emerge in biofilms; The expression of efflux pumps increases.

The formation of biomaterial-related biofilms mainly depends on the surface of medical devices and is a continuous, complex and multi-stage process that depends on many factors, such as the matrix, material surface medium, cell metabolism and internal characteristics, and signal molecules [13,14]. Biofilm formation includes three main steps: attachment, maturation, and dispersion [15,16] (Figure 1). In the first stage, bacteria attach to the biomaterial surface. Then, the bacteria form microcolonies when their adhesion to the biomaterial surface is stable. Finally, when the external conditions change, such as the temperature, nutrition, pH, or oxygen, the biofilm will disperse and the bacteria will return to the planktonic state [17,18].

### 2.1. Biofilm Initial Attachment

The initial adhesion stage of bacteria is not absolute due to the poor adhesion between bacterial cells and the biomaterial surface, but is instead a reversible process [14,19]. For example, when bacteria attach to a biomaterial surface, they are affected by the external environment, such as hydrodynamics and attraction or repulsion; or, if the composition, temperature, pressure, or pH of the biomaterial surface become unsuitable for the bacteria to adhere, the bacteria will return to the planktonic state [14,20].

In this process, physical interactions, such as polarity, London–van der Waals forces, electrostatic interactions, hydrophobic interactions, and protein adhesion, can promote the adhesion of microorganisms to the biomaterial surface and contribute to the formation of biofilms [14,21,22]. A flagellum is a flagellate filamentous appendage that provides power for bacteria. It can attach to the surface of biomaterials to enhance the interaction between the bacteria and the attached surface and can also reduce the repulsive force between the cells and the surface, thus promoting cell attachment to the surface [23,24]. Interestingly, under certain conditions, the relationship between bacteria and the biomaterial surface is transformed into one of irreversible attachment. First, bacterial cells are usually negatively charged, and when the attraction between the microorganism and the implanted surface is greater than the repulsive force, the bacterial cells remain fixed and strongly adhere to the positively charged surface to form irreversible adhesion [21]. Second, bacterial adhesin plays an important role in the adhesion of microorganisms to the biomaterial surface. Fimbriae adhesin allows bacteria to adhere to each other and form an initial cell adhesion layer. For example, type 1 fimbriae is one of the most common adhesion organelles in *Enterobacteriaceae*, including *Salmonella* [25]. It can increase the initial surface adhesion of *Escherichia coli* and form irreversible adhesion [26]. In addition, in some bacteria, polysaccharide adhesin can regulate permanent cell adhesion, such as polysaccharide intercellular adhesin (PIA), which promotes intercellular adhesion and biofilm accumulation of *S. epidermidis* [27,28]. Finally, EPSs can also promote the irreversible attachment of bacteria by interacting with surface materials and ligands [20].

Notably, adhesion is a key step for bacteria to form biofilms and cause persistent infections. Bacteria use adherens to stabilize colonization in the host [29]. For example, in immunocompromised cystic fibrosis patients, the main cause of persistent infection is the ability of *P. aeruginosa* to attach to the lungs and form biofilms [30]. Furthermore, *Burkholderia pseudomalei* can attach to host pharyngeal epithelial cells and cause respiratory infections [31], while attaching-effacing *E. coli* (AEEC) can adhere to intestinal mucosa through various mechanisms, resulting in intestinal infection [32]. In addition, bacteria can adhere to biological materials, which greatly promotes the production of biological fouling and material contamination of implanted medical devices, endangering human health [33].

### 2.2. Biofilm Maturation

After the microorganisms adhere to the biomaterial surface and stabilize, the bacterial cells will continue to proliferate and produce intercellular adhesions to form microcolonies [1]. At this stage, as the bacterial density reaches a threshold level, the bacterial quorum-sensing (QS) system is activated [21,34]. QS is a bacterial cell-to-cell communication system that relies on diffusible signal molecules. Bacteria use quorum-sensing signaling molecules to regulate their own behaviors, such as biofilm maturation, motility, and virulence factor expression [35,36]. Different types of bacteria secrete different signal molecules, such as acylated homoserine lactone (AHL) secreted by gram-negative bacteria, self-inducer peptide (AIP) secreted by gram-positive bacteria, and self-inducer-2 (AI-2) secreted by gram-negative and gram-positive bacteria [13,37]. Using *Pseudomonas aeruginosa* as an example, many studies have shown that *Pseudomonas* quinolone signal (PQS) molecules affect biofilm formation [38]. Deletion of the biosynthetic PQS gene caused *P. aeruginosa* to form a defective biofilm, and it could not form the mushroom-like structure of the mature biofilm of the wild bacteria [38]. Extracellular DNA (eDNA) is essential for intercellular junction and biofilm stability [21]. The PQS also promotes the release of eDNA, which is beneficial to the maturation of *P. aeruginosa* biofilms [39]. In addition, QS not only regulates the entire process of biofilm formation, but also promotes bacteria to secrete EPSs [40]. The secretion of EPSs helps to stabilize the biofilm structure and prevent it from being attacked by antibacterial agents and immune cells [41]. The secretion of EPSs also contributes to the formation of the three-dimensional structures of biofilm [42]. Voids and channels are formed in the three-dimensional structure of the biofilm, which are filled with water to distribute important nutrients, transmit molecular signals, remove waste from the biofilm, and act as a circulatory system [41,43]. After the formation of the first biofilm structure mediated by EPS, other bacteria of the same species and other species in the environment are also incorporated into the biofilm structure. The biofilm structure then develops from a thin layer into a mushroom-like or other three-dimensional structure [17,44,45,46].

### 2.3. Biofilm Dispersion

As the biofilm matures, nutrient resources are consumed in large quantities, and toxic substances will continue to accumulate [47,48]. Therefore, to obtain more nutrients, the bacterial cells of the biofilm disperse from the biomaterial surface and migrate to other areas of the medical implant, thereby spreading the infection [14,21]. The dispersion mechanism in bacteria generally occurs in three stages: first, cells leave the microcolony; second, the cells move to a new substrate; and third, the cells attach to the new substrate and start a new biofilm formation process [17,49]. In fact, biofilm dispersion can be divided into active and passive modes [50]. When bacteria experience environmental stresses, such as antimicrobial pressure and nutrient deficiency, they will actively dissociate from the implanted surface [51]. In the process of biofilm dispersion, some mechanisms are favorable for bacterial cell dispersion, such as the dissolution of EPSs [14]. Nutrient deprivation in biofilms stimulates small molecules of the fatty acid DSF (cis-11-methyl-2-dodecenoic acid) to trigger autophosphorylation and induce the degradation of c-di-GMP, leading to the dissolution of EPSs, which releases some of the planktonic cells [52,53]. In addition, in the process of active dispersion, the expression of genes related to bacterial motility caused by flagellum synthesis is upregulated, while the expression of genes related to bacterial attachment is downregulated, thus facilitating bacterial detachment [48].

The passive dispersion of biofilm depends on external factors, such as enzyme degradation of the biofilm matrix and shear forces [50]. The microbial community within the biofilm produces different saccharolytic enzymes that digest the polysaccharides that stabilize the biofilm structure, helping to release the microbial surfaces to new colonization areas [21,42]. For example, in alginate, Pel and Psl polysaccharides are the skeleton components of the *P. aeruginosa* biofilm-supporting structure, which can help bacteria absorb nutrients and communicate signals between cells [54]. Alginate lyases have been shown to be used for the cleavage and subsequent separation of EPS substrates, while the extracellular polysaccharide hydrolases PelA and PslG induce biofilm dispersion and prevent biofilm formation [21,55,56]. In one study, PelA was shown to be more effective in dispersing biofilms with Pel as the main extracellular polysaccharide, while PslG was most effective in removing biofilms formed with Psl as the main extracellular polysaccharide [57]. Previously, our research group also overexpressed PelA and PslG enzymes in *P. aeruginosa*-engineered bacteria through synthetic biology, and lysed the engineered bacteria in active and passive ways to release two types of extracellular polysaccharide hydrolases, PelA and PslG, thus destroying exoskeleton components Psl and Pel. The result was the destruction of the *P. aeruginosa* biofilm [58]. In addition to the enzymatic degradation of biofilm matrixes, physical means, such as shear force, are important factors for the passive dispersion of biofilms [59]. A sudden increase in shear forces causes the cells to be immediately released from the biofilm, resulting in the dispersion of the biofilm [60]. For example, during the formation of *Streptococcus mutans* biofilms, a 10-fold increase in shear forces resulted in an 85% reduction in the biomass of the biofilm [61]. In addition, ultrasonic waves, laser-induced shock waves, and other technologies have been found to passively disperse biofilms [50]. Ultrasonic treatment significantly reduced *E. coli* and *S. aureus* biofilms on a stainless-steel surface [62,63]. Laser-induced shock waves destroyed biofilms grown on different types of medical devices by generating plasma, and although the shock waves themselves were not harmful to the bacteria, the biofilms exposed to the shock waves were more sensitive to antibiotics than those that were not exposed [64]. To summarize, both active and passive methods can facilitate the dispersion of biofilm. However, once biofilm cells that were dispersed and settled on a new surface are stimulated by adverse conditions, the isolated microbial cells upregulate the expression of flagellar proteins to help them move quickly and return to a floating state on the surface. The bacteria then reform the biofilm in the right environment, causing continuous infection [14,65].

## 3. Mechanisms of Antibiotic-Resistant Biofilms

As one of the most successful life forms of bacteria, biofilms are not only widely distributed in various environments, but they also play an important role in promoting antibiotic resistance [11]. The sensitivity test of a biofilm model in vitro showed that the survival rate of bacteria covered by a biofilm was hundreds or even thousands of times higher than that of planktonic bacteria when using antibiotics for sterilization [66,67]. Many studies have shown that in biofilms, antibiotics do not exert their antibacterial function, but instead cause the emergence of superbugs, which increases the difficulty of treating biofilm infections [68,69]. In addition, the formation of a biofilm is conducive to the emergence of inflammation, which leads to persistent infection. For example, biofilm formation plays an important role in pulmonary infection, burn infection, and medical device infection in patients with cystic fibrosis caused by *P. aeruginosa* [70]. Therefore, revealing the main causes of drug resistance and the mechanism of drug resistance of biofilm bacteria is of great significance for the clinical treatment of biofilm infections [71].

### 3.1. Prevention of Antibiotic Penetration by Biofilms

EPSs not only maintain the structural stability of a biofilm, but also serve as a barrier for antimicrobial agents, such as bleaching agents and antibiotics [72]. Aminoglycoside antibiotics cannot penetrate the biofilm because the positive charge of the aminoglycoside antibiotics is neutralized by the negatively charged matrix [73]. For example, cations are chelated by a subinhibitory level of negatively charged eDNA, which limits the penetration of antibiotics by inducing the *PA3552–PA3559* operon to develop resistance to cationic antimicrobial peptides and aminoglycosides [71,74]. Furthermore, the presence of enzymes in the biofilm matrix also prevents the penetration of antibiotics [75]. For example, β-lactamases accumulated in extracellular polysaccharides promote the degradation and inactivation of antibiotics, and the role of β-lactamases is mainly against β-lactam antibiotics (carbapenem, imipenem), for which drug resistance is particularly important [76,77]. It is also believed that the poor permeability of antimicrobial agents is due to the decrease in diffusion and adsorption in the biofilm matrix [12], and there is no strong interaction between most antibiotics and biofilm matrix components [78,79].

### 3.2. Presence of Slow-Growing or Persistent Cells in Biofilms

The formation of the biofilm matrix leads to slower penetration of antimicrobial agents, causing the development of a resistant phenotype called persistent cells, resulting in antibiotic resistance [80,81]. An experiment of a biofilm in vitro showed that the oxygen concentration on the surface of a biofilm is high, and the metabolism of surface bacteria is also high, while the oxygen concentration in the center of the biofilm can be very low, and the bacteria growth is relatively slow or halted [76,82]. This is one of the reasons behind the decrease of biofilm sensitivity to antibiotics [81]. For example, fluoroquinolones and tetracycline can only effectively kill metabolically active cells in the upper layer of a *P. aeruginosa* biofilm [83]. Another reason for this decrease is the presence of persistent cells in biofilms [75]. Because the cell metabolism of persistent cells slows or ceases, the target of the antibiotic is not active, and the antibiotic cannot work [80]. In contrast to resistant mutants, persisters are phenotypic variants of wild-type cells, and it is worth noting that persisters are only transiently resistant to antibiotics.

### 3.3. Increased Expression of Efflux Pumps in Biofilms

Another possible mechanism of antibiotic resistance in biofilms is the increased expression of bacterial efflux pumps [84,85]. Efflux pumps, which are membrane proteins necessary to maintain bacterial homeostasis by expelling toxic substances, are present in all types of bacteria [86]. Bacteria present in the biofilms also use efflux pumps to maintain cytoplasmic concentrations of certain antibacterial compounds below critical thresholds or to expel antibiotics, and the antibiotic resistance of the biofilm is caused by the increased expression or activity of the efflux pumps [87,88]. The ATP binding box (ABC) family, the small multi-drug resistant (SMR) family, the multi-drug and toxic compound extrusion (MATE) family, the drug-resistant nodular cell division (RND) family, and the large promoter superfamily (MFS) are the five major families of efflux pumps, and they play an important role in biofilm antibiotic resistance [89,90]. For example, in *Acinetobacter baumannii*, TetA and TetB efflux pumps of the MFS family cause resistance of its biofilm to tetracycline and minocycline, in which the Tet efflux pump uses proton exchange as an energy source to extrude tetracycline [91]. The physiological function of these pumps is to expel toxic molecules that denature cell membranes and confer resistance to bacteria that are present in the biofilms [92]. MacB is a member of the ABC family, and it helps bacteria excrete macrolides and endows biofilms with antibiotic resistance [93]. In addition, *P. aeruginosa* MexAB-OprM and MexCD-OprJ, which belong to the RND family, play important roles in antibiotic resistance in biofilms, and MexAB-OprM pumps contribute to biofilm resistance to ofloxacin [94,95]. *S. aureus* responds to antibiotics by upregulating various ABC transporters, thereby increasing antibiotic resistance in biofilms. For example, GraRS may confer vancomycin resistance to a biofilm by upregulating the VraFGABC transporter [96,97]. BraRS can also activate BraDE and VraDEABC transporters and increase biofilm resistance to nisin and bacitracin [98,99].

## 4. Clinical Characteristics of Biomaterial-Associated Biofilms

Colonization of biomaterials by pathogens leads to the formation of biofilms, which greatly increases the potential for clinical infection [100]. This includes devices such as mechanical heart valves, prosthetic joints, endotracheal tubes, and contact lenses with biofilm-related infection [101] (Table 1). Once biofilms are formed on medical devices, eradicating microbes becomes extremely difficult and can be costly due to the lengthy hospital stays, surgery, and long-term antibacterial treatments that are often required [102]. The development of these biomaterials has saved millions of lives, but at the same time, the resulting biofilm infections are endangering people’s health. The above-mentioned associated biofilm infections caused by biomaterials share the same clinical features [103,104]:

(1) Persistent infection (more than 7 days): For example, central venous catheter-associated biofilms and almost all indwelling central venous catheters are colonized by microorganisms that can produce biofilms. Common bacteria isolated from catheter biofilms are coagulase-negative *Staphylococcus* [105]. Colonization and biofilm formation on catheter surfaces by these microorganisms can occur within 24 h of insertion and are pervasive and persistent [106]. In one study, short-term (<10 days) catheters had greater biofilm formation on the external surface; long-term catheters (>30 days) had more biofilm formation on the catheter inner lumen [42].
(2) Inflammation: An example is a biofilm infection associated with mechanical heart valves. Infectious endocarditis (IE) is a disease with substantial morbidity and mortality today due to the increasing use of implantable devices, such as prosthetic heart valves [107]. Microbes initially adhere to the prosthetic valve surface via fibronectin and polysaccharides and grow in a platelet–fibrin matrix to form an intact biofilm [108]. Because biofilms formed on artificial surfaces promote inflammatory responses and hypercoagulability, the presence of foreign tissue needs to be maintained through inflammatory and thrombotic processes [109]. In addition, prosthetic joint infection (PJI), a serious complication after joint replacement, causes inflammation [110]. According to the symptoms that appear after implantation, PJIs can be divided into three categories: early infection, delayed infection, and late infection [111]. Both early and delayed infections are often the result of surgical contamination and are considered the most common cause of biomaterial-related biofilm infections. These infections are often associated with local and systemic symptoms, in addition to triggering an inflammatory response that is accompanied by increases in laboratory markers of inflammation, such as C-reactive protein, erythrocyte sedimentation rate, and white blood cell count levels [112,113,114]. Therefore, in addition to routine methods for detecting biofilm-associated infections, blood and tissue cultures can be used to detect PJI infections at an early stage [113,115].
(3) Failure of antibiotic treatment and recurrence of infection: It is the formation of biofilms on the surface of biomaterials that leads to the development of drug resistance in the treatment of related infections [116]. The formation of a biofilm matrix can maintain temporary dormancy of the cells, which may lead to repeated infections [117]. For example, the treatment of PJI requires complex therapeutic strategies, and it is the long-term infection problems that lead to multiple surgical revisions and long-term antimicrobial therapy [110].

**Table 1:** Commonly reported infections in medical biomaterials.

Application	Biomaterials	Infection-Causing Agents	References
Orthopedic devices	Stainless steel, cobalt-based alloys, titanium, silicone, polyethylene, polypropylene, polymethyl methacrylate, aluminum oxide, and calcium phosphates	*S. epidermidis*, *S. aureus*, and *Staphylococcus hominis*	[118]
Prosthetic heart valves	Titanium, graphite, pyrolytic carbon, polyethylene, polypropylene, polyamide, and diamond-like carbon	*S. aureus*, *S. epidermidis*, *Candida albicans*, and *Aspergillus*	[119]
Dental implants	Acrylic resin, titanium and its alloys, zirconia, silver and silver nanoparticles, and ZnO	Gram-negative anaerobic bacteria (*Porphiromonas gengivalis*, *Actinobacillus actinomycetemcomitans*, *Fusobacterium nucleatum*, and *Veillonella* spp.)	[120]
Urologic devices	Polytetrafluoroethylene, rubber, polyurethane, polyamide, silicones, and polyhydroxyalkonates	*S. aureus* and *S. epidermis*	[121]
Bone allografts	Calcium phosphate ceramic, calcium sulphate, hydroxyapatite, bioactive glasses, and magnesium	*Hepatitis, tuberculosis*, and *human immunodeficiency virus*	[122,123]
Contact lenses and corneal implants	Silicone hydrogel and polymethylmethacrylate	*P. aeruginosa* and Gram-positive cocci	[124]
Breast implants	Silicone gel within silicone rubber envelopes and inflatable saline	*S. aureus*, *Enterococcus* spp., *S. epidermidis*, and *P. acnes*	[125,126,127]

## 5. Treatment of Biofilms on the Surface of Biomaterials

The biofilm that causes infection is progressive. Current treatments for implant-associated infections involve the administration of high doses of antibiotics and surgical replacement if symptoms persist [128]. The increase in antibiotic resistance has introduced problems in the treatment of patients using medical devices [129]. Therefore, several new biomaterials have been developed to treat clinical medical device-related biofilm infection, from the addition of antimicrobials to surface modifications of the material itself, which have been used as alternative strategies for the prevention and treatment of biofilm infections (Table 2).

### 5.1. Antibacterial Coatings

Antibacterial materials are widely used in the biomedical field, especially in the treatment of biofilm. The main pathogens of medical device-related infections are methicillin-resistant *S. aureus* (MRSA) and *E. coli* [148]. A common strategy for the treatment of this type of bacterial infection is antibiotic treatment. However, traditional antibiotic therapy will not only cause a variety of side effects, but is also more likely to cause the emergence of multi-drug-resistant bacteria [149]. As a result, new methods apart from antibiotic therapy have emerged to solve the problem of infections caused by medical devices, such as the use of antibacterial coatings on the surface of implants [150]. Implant coatings against biofilm-based infections can be divided into two categories [150]: (1) active coatings, which can fight infection by releasing pre-incorporated antimicrobial agents; the coatings are prepared by soaking a porous material or coating a surface with the desired antimicrobial compound, frequently using carriers such as hydroxyapatite (HA) and polycaprolactone (PCL) [151]; and (2) passive coatings, which prevent bacterial attachment through the use of hydrophilic materials [113].

#### 5.1.1. Active Coatings

The most common active coatings are antibiotic or silver compound coatings, which are applied to implant surfaces as antimicrobial or anti-biofilm agents for the prevention of medical device-related infections [152,153,154]. For example, the antibiotic hydroxyapatite coating, which is widely used in the medical field, can be used with antibiotic solutions, and modified by surface-adsorbed antibiotics, resulting in anti-biofilm effects [53]. Polymers usually have no intrinsic antibacterial properties, and polymer antibacterial materials can be prepared by using antibacterial additives to modify a polymer matrix in different ways [155]. For example, nanomaterials show unique biological and physicochemical properties, and their mode of action is believed to be the destruction of DNA through the formation of free radicals or the production of oxidative stress. The modes responsible for the antimicrobial activity of different NPs may comprise certain properties, such as composition, size, surface charge, and shape [156]. A study incorporated antibacterial Ag nanoparticles into a hydrophobic polymer in situ in a polycaprolactone matrix and achieved long-term release of active silver. The Ag-containing composite material had strong antibacterial and anti-biofilm properties and could therefore be used in medical antibacterial equipment [130]. Some zwitterionic polymers, such as hydrogels and sulfobetaine polymers, can delay or even prevent microorganisms from attaching to the surface because the tight hydration layer around the ion surface prevents the adsorption of non-specific proteins [157,158]. However, zwitterionic polymers cannot inactivate bacterial cells and, therefore, can be used as carriers to achieve synergistic antibacterial effects with fungicides [158]. Impregnating ultrasmall silver nanoparticles (AgNPs) into biocompatible thermosensitive hydrogels can not only control the delivery of AgNPs at an appropriate concentration, but also confers long-term storage stability and highly potent antibacterial activity [131]. AgNP hydrogels show excellent biofilm dispersion properties and are safe and non-toxic to mammalian cells in a wound environment, which provides a promising new strategy for hydrogels as an effective antibacterial platform [131]. In addition to traditional antibacterial agents, the release of other molecules (such as enzymes and antimicrobial peptides) can be used to treat infections. One study found that the acylase enzyme coating on the surface of a silicone catheter significantly inhibited the formation of a biofilm on its surface [132]. The negatively charged acylase enzymes were immobilized on the silicone catheter through alternate deposition, and the acylase enzymes on the surface reduced the biofilm by inducing the degradation of QS signals [132]. Antimicrobial peptides have been shown to effectively kill bacterial cells without toxic effects [159,160]. For example, a thin polymer multilayer film composed of chitosan and hyaluronic acid covered a catheter, and then a β-peptide coating was applied to the surface of the membrane. The film containing the peptide inhibited bacterial biofilm formation by releasing the β-peptide [133]. These β-peptide-containing films offer a new and promising localized delivery method for preventing orthopedic implant infections.

#### 5.1.2. Passive Coatings

Passive coatings are used to prevent bacteria from attaching. The development of coatings that prevent bacterial attachment is a promising approach to prevent biofilm formation using non-cytotoxic mechanisms [150]. The attachment of bacteria to the surface of biomaterials mainly consists of two key stages: the initial reversible attachment through physicochemical interactions and the irreversible attachment mediated by bacterial adhesion proteins [14]. Therefore, blocking the adhesion of bacteria on the surface of biomaterials provides a good theoretical target for the development of new therapeutic methods. Some strategies to reduce bacterial adhesion to biomaterial surfaces include the use of hydrophilic polymers, such as hyaluronic acid, hydrogel coatings, and heparin coatings [14]. Medical-related devices made of hydrophobic materials are more prone to biofilm infection due to the commonly observed preferential attachment of bacteria on hydrophobic surfaces [151,161]. In contrast, hydrophilic surfaces are covered by water molecules and prevent the attachment of cells and bacteria, which play roles as physical and energy barriers [162]. Previous studies found that hyaluronic acid has notable antiadhesive properties and shows moderate activity against bacterial biofilms [163]. A recent study combined quaternized chitosan with bactericidal properties, acylase with anti-quorum-sensing properties, and hyaluronic acid with anti-adhesion properties. This multifunctional coating inhibited most bacteria from initially attaching, killed the attached bacteria, and interfered with the quorum-sensing systems involved in biofilm formation [134]. In addition, chitosan can be used to prepare thermosensitive hydrogels for the treatment of bone defects. These hydrogels have attracted wide attention because they can successfully regenerate bone tissue without surgical intervention, thus reducing the invasiveness of treatment [135,136].

Due to the enormous burden of biomaterial-related infections (those associated with implants and catheters), the effectiveness of traditional antibiotic therapy is diminished, and bacterial resistance is increasing. At the same time, the transfer of antibacterial compounds onto the surfaces of biomaterials is also limited; therefore, the use of antibacterial coatings has become a new approach to prevent infection. The polymer-based composite materials composed of antibacterial coatings and polymer materials are easy to manufacture and have high biocompatibility and are therefore widely used [151]. However, there are still challenges, such as the lack of long-term performance and the fact that many antibacterial materials have only been studied in vitro without entering clinical research [150]. Therefore, follow-up studies should address these important challenges and enable these materials to make real progress in the biomedical field.

### 5.2. Modification of the Surface of Medical Implants

The physicochemical properties of the material surface of implants affect the adhesion behavior of cells on the material surface and the subsequent biofilm formation process [164]. These characteristics include electrostatic interactions and van der Waals forces between the material surface and cells; the surface energy and hydrophobicity of the material; the morphological characteristics of the material surface, such as roughness, surface composition, and topography; and functional chemical group modifications of the material surface [165]. Surface modification of biomaterials is a potential strategy to prevent bacteria from attaching and forming biofilms on the surface of materials [166]. According to the modification process, the surface modification of polymers can be divided into physical modifications and chemical modifications.

#### 5.2.1. Physical Modifications

Surface-treated polymers have been widely used with biomedical materials, including optimized modified hydrogels, nanomaterials, and phage materials [167,168]. The surface modification of polymers by physics is relatively simple, economical, and effective. The surface of the polymer is modified by physical methods to change its wettability, but the modification does not change the inherent chemical properties of the polymer [169]. Common modification methods include changing the surface roughness of the materials and preparing hydrophobic or superhydrophobic surfaces, which affect cell adhesion [164,166,170].

Superhydrophobic surfaces have antibacterial adhesion properties, as they naturally have nano- or microscale structures, in addition to the repulsion of water, which limits the access of bacteria to the superhydrophobic surface [171,172]. A femtosecond laser-induced surface structure is a large-scale nano- and microstructure formation technology that can effectively modify the optical, electrical, mechanical, and tribological properties of materials [173]. A surface treated by a femtosecond laser shows different surface structure on scales of the order of nano- and micrometers. Jalil et al. produced superhydrophobic surfaces on gold with femtosecond laser pulses, in which the original hydrophilic Au was transformed into a superhydrophobic surface and reduced the adhesion of *E. coli*. The physical inhibition of bacterial colonies and biofilm formation by this technique is a crucial step in reducing antibiotic resistant infections [137]. In addition, studies have designed flexible superhydrophobic surfaces decorated with copper hydroxide nanowires that lead to dual-scale roughness and superhydrophobicity. These nanowires are grown separately and transferred onto polydimethylsiloxane (PDMS) surfaces by mechanical peeling; thus, non-planar 3D surfaces can be fabricated [138]. These surfaces have shown blood repellence, antibacterial activity, and hemocompatibility, indicating that they are suitable for medical and healthcare applications. In recent years, a novel surface has been produced that is characterized by double etching (DAE), which is a common method for the preparation of titanium surfaces in the production of dental implants [139]. Bacterial colonization of dental implants begins directly after exposure to the oral environment, where oral *Streptococcus oralis* is the initial colonizer of human plaque. This bacteria adheres to the acquired pellicle of soft and hard tissues in the oral cavity, creating preconditions for the adhesion of late colonizers [174]. The aim of DAE treatment is to create overlapping roughness at the nanometer and submicron ranges [175]; this not only changes the superficial topography and increases hydrophilicity, but it also increases the percentage of oxygen in the superficial layer, which may help inhibit oral *S. oralis* adhesion and biofilm formation [139]. Polyetheretherketone (PEEK), a novel material recently introduced in implantology, exhibits high nano-roughness [176]. A microbiological analysis measured the colony-forming units (CFUs), the biomass (OD_570_ detection), and the cell viability 24 and 48 h after *S. oralis* cultivation on the different discs; the results show that the PEEK surface-attached biomass and number of living cells were significantly reduced compared to the CFU on a double-etched titanium surface [140]. Therefore, the anti-adhesion and antibacterial properties of PEEK against pioneers such as *S. oralis* may play an important role in preventing the pathological aspects of biofilm formation, such as peri-implantitis in dentistry [120].

A change of roughness will change the surface energy and hydrophobicity of a material [177], which will affect cell adhesion. It is generally believed that a lower surface roughness reduces bacterial adhesion to commonly used implant materials and prevents the formation of biofilms on the biomaterial surfaces [178,179,180]. For example, etching technology is used to design micro- or nano-scale surface structures to increase the surface roughness of biomaterials [181,182]. Studies have used a simple etching method to produce multi-scale roughness on the surface of Al alloys. This method not only rapidly etched large-size substrates, but also showed a high bactericidal effect on rod-shaped and coccoid-shaped bacterial cells [141]. The latest advances in surface technology and materials have led to the development of surface patterns, some of which can affect bacterial colonization and biofilm formation without added antimicrobial agents, such as sharklet micropatterns that have been shown to control the bioadhesion of pathogenic bacteria [142,143]. Studies have incorporated nano- and shark micropatterns into the surface of central venous catheters. This surface modification reduced microbial colonization and biofilm formation and can be applied clinically to reduce catheter-related infection [144]. In addition, some studies have combined chemical and topographic properties to obtain a sharkskin-mimicking graphene oxide (GO)-modified chitosan membrane with antibacterial properties and cytocompatibility. GO-coated sharkskin-mimicking membranes can significantly reduce the formation of bacterial biofilms while promoting cytocompatibility, and can therefore be used as a convenient biomaterial in many biomedical applications, such as those requiring a biodegradable, highly biocompatible, and antibacterial implantable medical equipment coating [145].

Unlike other surface treatment methods, physical modifications are performed as part of the surface manufacturing itself. In addition, polymers modified by physical methods are strong and do not age or deteriorate, as the resulting surface roughness rarely affects the surface chemistry of the polymers [183,184].

#### 5.2.2. Chemical Modification

The initial adhesion of microorganisms and the formation of biofilms can be regulated by changing the chemical properties of the material surface [185]. The main methods of surface chemical modifications include the covalent modification of materials, non-covalent modifications, controlled release of small molecules, and degradation of the polymerized surface [186,187].

A commonly used chemical modification technique is the self-assembled monolayer (SAM), which is a thermodynamically stable and regularly arranged monolayer formed spontaneously by a physicochemical interaction between the molecules and the substrate materials [188,189]. SAM technology can effectively control the types of microbial-oriented functional groups and the concentration of ligands loaded on the material surface, and then purposefully change the surface energy and surface charge density [190]. For example, chitosan inhibits bacterial growth through the interaction between positively charged amino groups and negatively charged outer membranes [191]. It was shown that chitosan hydrogel films were covalently attached to thiol-modified substrates via a thiol one-click reaction. Afterward, heparin-mimicking polymer chains were grafted onto the hydrogel thin film layer via surface-initiated atom transfer radical polymerization, and covalent adhesion of the hydrogel was realized [146]. This self-defense double-layer hydrogel coating can switch from a cell adhesion surface to an antibacterial adhesion surface and provides a new approach for the antibacterial protection of biomedical equipment.

There are also several functional chemical modifications designed to work against the formation mechanism of bacterial biofilm, such as quorum-sensing inhibitors (QSIs) to inhibit the key regulatory mechanism of bacterial biofilm formation [192,193]. Surface-immobilized QSIs block bacterial communication and affect the formation of biofilms on the surface of biomaterials [194,195]. It has been found that furanone is a substance with a similar structure to N-acyl homoserine lactone (AHL), a signal molecule of gram-negative bacterial QS, which competes with AHLs for receptors, thus interfering with QS to inhibit biofilms [196]. The intergeneric quorum-sensing signal molecule autoinducer-2 (AI-2) of *F. nucleatum* is very important for the development of pathogenicity and biofilm formation [197]. A new brominated furanone was recently found that inhibited biofilm formation through AI-2, thus inhibiting biofilms caused by periodontopathogens, including *F. nucleatum*, *Porphyromonasgingivalis*, and *Tannerella forsythia* [147].

The physicochemical interaction between the material surface and cell provides a design idea for the preparation of materials that regulate microbial film formation. Through a series of complex physical, chemical, or biological mechanisms, the surface of the material has an impact on bacterial adhesion and the formation of biofilms. There are various methods for influencing these mechanisms, including the addition of an antibacterial coating on the material surface and the surface modification of biological materials. The surface properties of materials are very important to the research and development of inhibitory biofilm-forming materials, and the method of regulating biofilms through material modification provides a more comprehensive scientific reference for related research.

## 6. Conclusions

Biofilm formation can lead to the development of drug resistance in bacteria and lead to serious nosocomial infections. In particular, biomaterial-related biofilm infections are a major challenge for human health, and biofilm formation on medical devices is a major threat to medical device operation and public health. In response to this problem, conventional antibiotic therapy has been unable to effectively eradicate biofilms. In contrast, with the use of antibiotics, bacteria have evolved multiple resistance mechanisms, increasing the difficulty of treating biomaterial-associated biofilm infections. Therefore, it is of great practical significance to purposefully regulate the adhesion of microorganisms on material surfaces and the formation of biofilms. The use of antibacterial coatings or surface modifications of medical materials not only prevents bacterial adhesion, but also directly fights biofilm infections by releasing antibacterial drugs. In addition, appropriate antibacterial therapy can save money and shorten the duration of treatment.

With the increasing number of biomaterial studies of biofilm regulation, most of which are in vitro studies, more mechanistic studies are needed in the future to overcome these shortcomings and enable these therapies to enter clinical trials. To better solve the problem of biofilm resistance, it is necessary to conduct in-depth research and optimize preventive measures, such as developing new antibacterial coatings (biodegradable, biocompatible) and optimizing medical materials. In conclusion, the research in the field of biofilms involves the intersection and integration of multidisciplinary methods, such as microbiology, molecular biology, surface chemistry, and material science, which is not only of academic significance, but also of extremely important application value for clinical treatment, which still needs further research.

## Figures and Tables

**Table 2:** Different approaches to treat infectious biofilms on biomaterials.

Types		Mechanisms	Materials Preparation	References
Antibacterial coatings	Active coatings	Fight infection by releasing antimicrobial agents	Antibiotic or silver compound coatings, enzymes, antimicrobial peptides	[53,130,131,132,133]
	Passive coatings	Prevent bacteria from attaching using hydrophilic materials	Hyaluronic acid, hydrogel coatings	[134,135,136]
Modification of the surface of medical implants	Physical modification	Affect cell adhesion by changing the surface roughness and generating hydrophobic surfaces	Femtosecond laser-induced surfaces, superhydrophobic non-planar 3D surfaces, double-etched suface, polyetheretherketone, nano- and shark micropattern surfaces	[137,138,139,140,141,142,143,144,145]
	Chemical modification	Regulate the formation of biofilms by changing the chemical properties of the material surface	Chitosan hydrogel films, surface-immobilized brominated furanones	[146,147]

## Data Availability

Not applicable.

## References

[1] Costerton J.W., Stewart P.S., Greenberg E.P. (1999). Bacterial biofilms: A common cause of persistent infections. Science.

[2] Yan Z., Huang M., Melander C., Kjellerup B.V. (2020). Dispersal and inhibition of biofilms associated with infections. J. Appl. Microbiol..

[3] Assefa M., Amare A. (2022). Biofilm-associated multi-drug resistance in hospital-acquired infections: A review. Infect. Drug Resist..

[4] Tran H.M., Tran H., Booth M.A., Fox K.E., Nguyen T.H., Tran N., Tran P.A. (2020). Nanomaterials for treating bacterial biofilms on implantable medical devices. Nanomaterials.

[5] Zhang X., de Boer L., Stockhammer O.W., Grijpma D.W., Spaink H.P., Zaat S.A.J. (2019). A zebrafish embryo model for in vivo visualization and intravital analysis of biomaterial-associated *staphylococcus aureus* Infection. J. Vis. Exp..

[6] Rosman C.W.K., van Dijl J.M., Sjollema J. (2022). Interactions between the foreign body reaction and *Staphylococcus aureus* biomaterial-associated infection. Winning strategies in the derby on biomaterial implant surfaces. Crit. Rev. Microbiol..

[7] Vazquez-Rodriguez J.A., Shaqour B., Guarch-Pérez C., Choińska E., Riool M., Verleije B., Beyers K., Costantini V.J.A., Święszkowski W., Zaat S.A.J. (2022). A niclosamide-releasing hot-melt extruded catheter prevents *Staphylococcus aureus* experimental biomaterial-associated infection. Sci. Rep..

[8] Gebreyohannes G., Nyerere A., Bii C., Sbhatu D.B. (2019). Challenges of intervention, treatment, and antibiotic resistance of biofilm-forming microorganisms. Heliyon.

[9] Cao H., Qiao S., Qin H., Jandt K.D. (2022). Antibacterial designs for implantable medical devices: Evolutions and challenges. J. Funct. Biomater..

[10] Premkumar A., Kolin D.A., Farley K.X., Wilson J.M., McLawhorn A.S., Cross M.B., Sculco P.K. (2021). Projected economic burden of periprosthetic joint infection of the hip and knee in the united states. J. Arthroplast..

[11] Luo Y., Yang Q., Zhang D., Yan W. (2021). Mechanisms and control strategies of antibiotic resistance in pathological biofilms. J. Microbiol. Biotechnol..

[12] Mishra S., Gupta A., Upadhye V., Singh S.C., Sinha R.P., Häder D.P. (2023). Therapeutic strategies against biofilm infections. Life.

[13] Liu X., Yao H., Zhao X., Ge C. (2023). Biofilm formation and control of foodborne pathogenic bacteria. Molecules.

[14] Khatoon Z., McTiernan C.D., Suuronen E.J., Mah T.F., Alarcon E.I. (2018). Bacterial biofilm formation on implantable devices and approaches to its treatment and prevention. Heliyon.

[15] Wang D., Fletcher G.C., On S.L.W., Palmer J.S., Gagic D., Flint S.H. (2023). Biofilm formation, sodium hypochlorite susceptibility and genetic diversity of *Vibrio parahaemolyticus*. Int. J. Food Microbiol..

[16] Sauer K., Stoodley P., Goeres D.M., Hall-Stoodley L., Burmølle M., Stewart P.S., Bjarnsholt T. (2022). The biofilm life cycle: Expanding the conceptual model of biofilm formation. Nat. Rev. Microbiol..

[17] Öztürk F.Y., Darcan C., Kariptaş E. (2023). The determination, monitoring, molecular mechanisms and formation of biofilm in *E. coli*. Braz. J. Microbiol..

[18] Rabin N., Zheng Y., Opoku-Temeng C., Du Y., Bonsu E., Sintim H.O. (2015). Biofilm formation mechanisms and targets for developing antibiofilm agents. Future Med. Chem..

[19] De Silva L., Heo G.J. (2022). Biofilm formation of pathogenic bacteria isolated from aquatic animals. Arch. Microbiol..

[20] Abebe G.M. (2020). The role of bacterial biofilm in antibiotic resistance and food contamination. Int. J. Microbiol..

[21] Gupta P., Sarkar S., Das B., Bhattacharjee S., Tribedi P. (2016). Biofilm, pathogenesis and prevention—A journey to break the wall: A review. Arch. Microbiol..

[22] Stoica P., Chifiriuc M.C., Rapa M., Lazăr V. (2017). Overview of biofilm-related problems in medical devices. Biofilms and Implantable Medical Devices.

[23] Li P., Zong W., Zhang Z., Lv W., Ji X., Zhu D., Du X., Wang S. (2023). Effects and molecular mechanism of flagellar gene flgK on the motility, adhesion/invasion, and desiccation resistance of *Cronobacter sakazakii*. Food Res. Int..

[24] Nedeljković M., Sastre D.E., Sundberg E.J. (2021). Bacterial flagellar filament: A supramolecular multifunctional nanostructure. Int. J. Mol. Sci..

[25] Kolenda R., Ugorski M., Grzymajlo K. (2019). Everything you always wanted to know about *salmonella* type 1 fimbriae, but were afraid to ask. Front. Microbiol..

[26] Puri D., Fang X., Allison K.R. (2023). Evidence of a possible multicellular life cycle in *Escherichia coli*. iScience.

[27] Azara E., Longheu C.M., Attene S., Sanna S., Sale M., Addis M.F., Tola S. (2022). Comparative profiling of agr locus, virulence, and biofilm-production genes of human and ovine non-aureus staphylococci. BMC Vet. Res..

[28] Mack D., Fischer W., Krokotsch A., Leopold K., Hartmann R., Egge H., Laufs R. (1996). The intercellular adhesin involved in biofilm accumulation of *Staphylococcus epidermidis* is a linear beta-1,6-linked glucosaminoglycan: Purification and structural analysis. J. Bacteriol..

[29] Lv Z.T., Qian C., Liu Y.N., Lv Y.H., Liu X.W. (2022). Optical tracking of surfactant-tuned bacterial adhesion: A single-cell imaging study. Appl. Environ. Microbiol..

[30] Pellielo G., Agyapong E.D., Pinton P., Rimessi A. (2023). Control of mitochondrial functions by *Pseudomonas aeruginosa* in cystic fibrosis. Int. Rev. Cell Mol. Biol..

[31] Mariappan V., Thimma J., Vellasamy K.M., Shankar E.M., Vadivelu J. (2018). Adhesion and invasion attributes of *Burkholderia pseudomallei* are dependent on airway surface liquid and glucose concentrations in lung epithelial cells. Environ. Microbiol. Rep..

[32] Wales A.D., Woodward M.J., Pearson G.R. (2005). Attaching-effacing bacteria in animals. J. Comp. Pathol..

[33] Pierrat X., Wong J.P.H., Al-Mayyah Z., Persat A. (2021). The mammalian membrane microenvironment regulates the sequential attachment of bacteria to host cells. mBio.

[34] Federle M.J., Bassler B.L. (2003). Interspecies communication in bacteria. J. Clin. Investig..

[35] Xiao Y., Zou H., Li J., Song T., Lv W., Wang W., Wang Z., Tao S. (2022). Impact of quorum sensing signaling molecules in gram-negative bacteria on host cells: Current understanding and future perspectives. Gut Microbes..

[36] Lin J., Cheng J., Wang Y., Shen X. (2018). The *Pseudomonas* Quinolone Signal (PQS): Not just for quorum sensing anymore. Front. Cell Infect. Microbiol..

[37] Wang Y., Bian Z., Wang Y. (2022). Biofilm formation and inhibition mediated by bacterial quorum sensing. Appl. Microbiol. Biotechnol..

[38] Cooke A.C., Florez C., Dunshee E.B., Lieber A.D., Terry M.L., Light C.J., Schertzer J.W. (2020). *Pseudomonas* Quinolone Signal-induced outer membrane vesicles enhance biofilm dipersion in *Pseudomonas aeruginosa*. mSphere.

[39] Allesen-Holm M., Barken K.B., Yang L., Klausen M., Webb J.S., Kjelleberg S., Molin S., Givskov M., Tolker-Nielsen T. (2006). A characterization of DNA release in *Pseudomonas aeruginosa* cultures and biofilms. Mol. Microbiol..

[40] Fu H., Wang J., Liu Q., Ding L., Ren H. (2022). The role of immobilized quorum sensing strain in promoting biofilm formation of Moving Bed Biofilm Reactor during long-term stable operation. Environ. Res..

[41] Yin R., Cheng J., Wang J., Li P., Lin J. (2022). Treatment of *Pseudomonas aeruginosa* infectious biofilms: Challenges and strategies. Front. Microbiol..

[42] Jamal M., Ahmad W., Andleeb S., Jalil F., Imran M., Nawaz M.A., Hussain T., Ali M., Rafiq M., Kamil M.A. (2018). Bacterial biofilm and associated infections. J. Chin. Med. Assoc..

[43] Parsek M.R., Singh P.K. (2003). Bacterial biofilms: An emerging link to disease pathogenesis. Annu. Rev. Microbiol..

[44] Wimpenny J., Manz W., Szewzyk U. (2000). Heterogeneity in biofilms. FEMS Microbiol. Rev..

[45] Schilcher K., Horswill A.R. (2020). Staphylococcal Biofilm Development: Structure, Regulation, and Treatment Strategies. Microbiol. Mol. Biol. Rev..

[46] Morelli K.A., Kerkaert J.D., Cramer R.A. (2021). *Aspergillus fumigatus* biofilms: Toward understanding how growth as a multicellular network increases antifungal resistance and disease progression. PLoS Pathog..

[47] Sauer K., Cullen M.C., Rickard A.H., Zeef L.A., Davies D.G., Gilbert P. (2004). Characterization of nutrient-induced dispersion in *Pseudomonas aeruginosa* PAO1 biofilm. J. Bacteriol..

[48] Kostakioti M., Hadjifrangiskou M., Hultgren S.J. (2013). Bacterial biofilms: Development, dispersal, and therapeutic strategies in the dawn of the postantibiotic era. Cold Spring Harb. Perspect. Med..

[49] Shen D., Langenheder S., Jürgens K. (2018). Dispersal modifies the diversity and composition of active bacterial communities in response to a salinity disturbance. Front. Microbiol..

[50] Wille J., Coenye T. (2020). Biofilm dispersion: The key to biofilm eradication or opening Pandora’s box?. Biofilm.

[51] Fleming D., Rumbaugh K.P. (2017). Approaches to dispersing medical biofilms. Microorganisms.

[52] Oppenheimer-Shaanan Y., Steinberg N., Kolodkin-Gal I. (2013). Small molecules are natural triggers for the disassembly of biofilms. Trends Microbiol..

[53] Veerachamy S., Yarlagadda T., Manivasagam G., Yarlagadda P.K. (2014). Bacterial adherence and biofilm formation on medical implants: A review. Proc. Inst. Mech. Eng. H.

[54] Ma L.Z., Wang D., Liu Y., Zhang Z., Wozniak D.J. (2022). Regulation of biofilm exopolysaccharide biosynthesis and degradation in *Pseudomonas aeruginosa*. Annu. Rev. Microbiol..

[55] Sutherland I.W. (1999). Polysaccharases for microbial exopolysaccharides. Carbohydr. Polym..

[56] Kalia M., Resch M.D., Cherny K.E., Sauer K. (2022). The alginate and motility regulator AmrZ is essential for the regulation of the dispersion response by *Pseudomonas aeruginosa* biofilms. mSphere.

[57] Colvin K.M., Irie Y., Tart C.S., Urbano R., Whitney J.C., Ryder C., Howell P.L., Wozniak D.J., Parsek M.R. (2012). The Pel and Psl polysaccharides provide *Pseudomonas aeruginosa* structural redundancy within the biofilm matrix. Environ. Microbiol..

[58] Wang S.T., Niu Y.T., Zhang H., Li P.X., Zhang N.M., Cheng J.L., Lin J.S. (2021). An engineered bacterium for the targeted delivery of proteins to destroy *Pseudomonas aeruginosa* biofilms. Acta Microbiol. Sin..

[59] Tsagkari E., Connelly S., Liu Z., McBride A., Sloan W.T. (2022). The role of shear dynamics in biofilm formation. NPJ Biofilms Microbiomes.

[60] Choi Y.C., Morgenroth E. (2003). Monitoring biofilm detachment under dynamic changes in shear stress using laser-based particle size analysis and mass fractionation. Water Sci. Technol..

[61] Hwang G., Klein M.I., Koo H. (2014). Analysis of the mechanical stability and surface detachment of mature *Streptococcus mutans* biofilms by applying a range of external shear forces. Biofouling.

[62] Oulahal-Lagsir N., Martial-Gros A., Bonneau M., Blum L.J. (2003). “*Escherichia coli*-milk” biofilm removal from stainless steel surfaces: Synergism between ultrasonic waves and enzymes. Biofouling.

[63] Oulahal N., Martial-Gros A., Bonneau M., Blum L.J. (2004). Combined Effect of Chelating Agents and Ultrasound on Biofilm Removal from Stainless Steel Surfaces. Application to Escherichia coli Milk and Staphylococcus aureus Milk Biofilms.

[64] Nigri G.R., Tsai S., Kossodo S., Waterman P., Fungaloi P., Hooper D.C., Doukas A.G., LaMuraglia G.M. (2001). Laser-induced shock waves enhance sterilization of infected vascular prosthetic grafts. Lasers Surg. Med..

[65] Otto M. (2013). *Staphylococcal* infections: Mechanisms of biofilm maturation and detachment as critical determinants of pathogenicity. Annu. Rev. Med..

[66] Ceri H., Olson M.E., Stremick C., Read R.R., Morck D., Buret A. (1999). The calgary biofilm device: New technology for rapid determination of antibiotic susceptibilities of bacterial biofilms. J. Clin. Microbiol..

[67] Michael, Waturangi D.E. (2023). Antibiofilm activity from endophyte bacteria, *Vibrio cholerae* strains, and actinomycetes isolates in liquid and solid culture. BMC Microbiol..

[68] Pokharel K., Dawadi B.R., Shrestha L.B. (2022). Role of biofilm in bacterial infection and antimicrobial resistance. JNMA J. Nepal. Med. Assoc..

[69] Cascioferro S., Carbone D., Parrino B., Pecoraro C., Giovannetti E., Cirrincione G., Diana P. (2021). Therapeutic strategies to counteract antibiotic resistance in MRSA biofilm-associated infections. ChemMedChem.

[70] Thomsen K., Høiby N., Jensen P., Ciofu O., Moser C. (2022). Immune response to biofilm growing pulmonary *Pseudomonas aeruginosa* infection. Biomedicines.

[71] Güneş B., Akçelik N. (2022). The role of eDNA in biofilm structure of *Enterococcus faecalis* and investigation of the efficiency of enzyme and antibiotic application in biofilm eradication. Mikrobiyol. Bul..

[72] Sheng Y., Chen Z., Wu W., Lu Y. (2023). Engineered organic nanoparticles to combat biofilms. Drug Discov. Today.

[73] Tian C., Yuan M., Tao Q., Xu T., Liu J., Huang Z., Wu Q., Pan Y., Zhao Y., Zhang Z. (2023). Discovery of novel resistance mechanisms of *Vibrio parahaemolyticus* biofilm against aminoglycoside antibiotics. Antibiotics.

[74] Mulcahy H., Charron-Mazenod L., Lewenza S. (2008). Extracellular DNA chelates cations and induces antibiotic resistance in *Pseudomonas aeruginosa* biofilms. PLoS Pathog..

[75] Ciofu O., Rojo-Molinero E., Macià M.D., Oliver A. (2017). Antibiotic treatment of biofilm infections. Apmis.

[76] Hoiby N., Bjarnsholt T., Givskov M., Molin S., Ciofu O. (2010). Antibiotic resistance of bacterial biofilms. Int. J. Antimicrob. Agents.

[77] Mohamed H.M.A., Alnasser S.M., Abd-Elhafeez H.H., Alotaibi M., Batiha G.E., Younis W. (2022). detection of β-Lactamase resistance and biofilm genes in *Pseudomonas* species isolated from chickens. Microorganisms.

[78] Yan J., Moreau A., Khodaparast S., Perazzo A., Feng J., Fei C., Mao S., Mukherjee S., Košmrlj A., Wingreen N.S. (2018). Bacterial biofilm material properties enable removal and transfer by capillary peeling. Adv. Mater..

[79] Spoering A.L., Lewis K. (2001). Biofilms and planktonic cells of *Pseudomonas aeruginosa* have similar resistance to killing by antimicrobials. J. Bacteriol..

[80] Mah T.F. (2012). Biofilm-specific antibiotic resistance. Future Microbiol..

[81] Stewart P.S. (2002). Mechanisms of antibiotic resistance in bacterial biofilms. Int. J. Med. Microbiol..

[82] Costerton J.W., Lewandowski Z., Caldwell D.E., Korber D.R., Lappin-Scott H.M. (1995). Microbial biofilms. Annu. Rev. Microbiol..

[83] Pamp S.J., Gjermansen M., Johansen H.K., Tolker-Nielsen T. (2008). Tolerance to the antimicrobial peptide colistin in *Pseudomonas aeruginosa* biofilms is linked to metabolically active cells, and depends on the pmr and mexAB-oprM genes. Mol. Microbiol..

[84] Gaurav A., Bakht P., Saini M., Pandey S., Pathania R. (2023). Role of bacterial efflux pumps in antibiotic resistance, virulence, and strategies to discover novel efflux pump inhibitors. Microbiology.

[85] Seukep A.J., Mbuntcha H.G., Kuete V., Chu Y., Fan E., Guo M.Q. (2022). What approaches to thwart bacterial efflux pumps-mediated resistance?. Antibiotics.

[86] Başaran S.N., Öksüz L. (2023). The role of efflux pumps ın antıbıotıc resıstance of gram negatıve rods. Arch. Microbiol..

[87] Mudde S.E., Schildkraut J.A., Ammerman N.C., de Vogel C.P., de Steenwinkel J.E.M., van Ingen J., Bax H.I. (2022). Unraveling antibiotic resistance mechanisms in *Mycobacterium abscessus*: The potential role of efflux pumps. J. Glob. Antimicrob. Resist..

[88] Barnabas V., Kashyap A., Raja R., Newar K., Rai D., Dixit N.M., Mehra S. (2022). The extent of antimicrobial resistance due to efflux pump regulation. ACS Infect. Dis..

[89] Reygaert W.C. (2018). An overview of the antimicrobial resistance mechanisms of bacteria. AIMS Microbiol..

[90] Uddin T.M., Chakraborty A.J., Khusro A., Zidan B.R.M., Mitra S., Emran T.B., Dhama K., Ripon M.K.H., Gajdács M., Sahibzada M.U.K. (2021). Antibiotic resistance in microbes: History, mechanisms, therapeutic strategies and future prospects. J. Infect. Public Health.

[91] Grossman T.H. (2016). Tetracycline antibiotics and resistance. Cold Spring Harb. Perspect. Med..

[92] Venkatesan N., Perumal G., Doble M. (2015). Bacterial resistance in biofilm-associated bacteria. Future Microbiol..

[93] Shi K., Cao M., Li C., Huang J., Zheng S., Wang G. (2019). Efflux proteins MacAB confer resistance to arsenite and penicillin/macrolide-type antibiotics in *Agrobacterium tumefaciens* 5A. World J. Microbiol. Biotechnol..

[94] Ahmadian L., Haghshenas M.R., Mirzaei B., Khalili Y., Goli H.R. (2023). Role of MexAB-OprM efflux pump in the emergence of multidrug-resistant clinical isolates of *Pseudomonas aeruginosa* in Mazandaran province of Iran. Mol. Biol. Rep..

[95] Ferrer-Espada R., Shahrour H., Pitts B., Stewart P.S., Sánchez-Gómez S., Martínez-de-Tejada G. (2019). A permeability-increasing drug synergizes with bacterial efflux pump inhibitors and restores susceptibility to antibiotics in multi-drug resistant *Pseudomonas aeruginosa* strains. Sci. Rep..

[96] Yang S.J., Bayer A.S., Mishra N.N., Meehl M., Ledala N., Yeaman M.R., Xiong Y.Q., Cheung A.L. (2012). The *Staphylococcus aureus* two-component regulatory system, GraRS, senses and confers resistance to selected cationic antimicrobial peptides. Infect. Immun..

[97] Cho J., Costa S.K., Wierzbicki R.M., Rigby W.F.C., Cheung A.L. (2021). The extracellular loop of the membrane permease VraG interacts with GraS to sense cationic antimicrobial peptides in *Staphylococcus aureus*. PLoS Pathog..

[98] Hiron A., Falord M., Valle J., Débarbouillé M., Msadek T. (2011). Bacitracin and nisin resistance in *Staphylococcus aureus*: A novel pathway involving the BraS/BraR two-component system (SA2417/SA2418) and both the BraD/BraE and VraD/VraE ABC transporters. Mol. Microbiol..

[99] Arii K., Kawada-Matsuo M., Oogai Y., Noguchi K., Komatsuzawa H. (2019). Single mutations in BraRS confer high resistance against nisin A in *Staphylococcus aureus*. Microbiologyopen.

[100] Babushkina I.V., Bondarenko A.S., Ulyanov V.Y., Mamonova I.A. (2020). Biofilm formation by gram-negative bacteria during implant-associated infection. Bull. Exp. Biol. Med..

[101] Barros J., Monteiro F.J., Ferraz M.P. (2022). Bioengineering approaches to fight against orthopedic biomaterials related-infections. Int. J. Mol. Sci..

[102] Chouirfa H., Bouloussa H., Migonney V., Falentin-Daudré C. (2019). Review of titanium surface modification techniques and coatings for antibacterial applications. Acta Biomater..

[103] Høiby N., Bjarnsholt T., Moser C., Bassi G.L., Coenye T., Donelli G., Hall-Stoodley L., Holá V., Imbert C., Kirketerp-Møller K. (2015). ESCMID guideline for the diagnosis and treatment of biofilm infections 2014. Clin. Microbiol. Infect..

[104] Wi Y.M., Patel R. (2018). Understanding biofilms and novel approaches to the diagnosis, prevention, and treatment of medical devicea-Associated infections. Infect. Dis. Clin. N. Am..

[105] Lebeaux D., Fernández-Hidalgo N., Chauhan A., Lee S., Ghigo J.M., Almirante B., Beloin C. (2014). Management of infections related to totally implantable venous-access ports: Challenges and perspectives. Lancet Infect. Dis..

[106] Gominet M., Compain F., Beloin C., Lebeaux D. (2017). Central venous catheters and biofilms: Where do we stand in 2017?. Apmis.

[107] Mangner N., Woitek F., Haussig S., Schlotter F., Stachel G., Höllriegel R., Wilde J., Lindner A., Holzhey D., Leontyev S. (2016). Incidence, predictors, and outcome of patients developing infective endocarditis following transfemoral transcatheter aortic valve replacement. J. Am. Coll. Cardiol..

[108] Elgharably H., Hussain S.T., Shrestha N.K., Blackstone E.H., Pettersson G.B. (2016). Current hypotheses in cardiac surgery: Biofilm in infective endocarditis. Semin. Thorac. Cardiovasc. Surg..

[109] Nappi F., Iervolino A., Singh S.S.A. (2021). The new challenge for heart endocarditis: From conventional prosthesis to new devices and platforms for the treatment of structural heart disease. Biomed. Res. Int..

[110] Izakovicova P., Borens O., Trampuz A. (2019). Periprosthetic joint infection: Current concepts and outlook. EFORT Open Rev..

[111] Kamihata S., Ando W., Nakahara I., Enami H., Takashima K., Uemura K., Hamada H., Sugano N. (2023). Optimizing vancomycin release from novel carbon fiber-reinforced polymer implants with small holes: Periprosthetic joint infection treatment. J. Artif. Organs.

[112] Belgiovine C., Pellegrino L., Bulgarelli A., Lauta F.C., Di Claudio A., Ciceri R., Cancellara A., Calcaterra F., Mavilio D., Grappiolo G. (2023). Interaction of bacteria, immune cells, and surface topography in periprosthetic joint infections. Int. J. Mol. Sci..

[113] Gbejuade H.O., Lovering A.M., Webb J.C. (2015). The role of microbial biofilms in prosthetic joint infections. Acta Orthop..

[114] Osmon D.R., Berbari E.F., Berendt A.R., Lew D., Zimmerli W., Steckelberg J.M., Rao N., Hanssen A., Wilson W.R. (2013). Diagnosis and management of prosthetic joint infection: Clinical practice guidelines by the Infectious Diseases Society of America. Clin. Infect. Dis..

[115] Jacqueline C., Caillon J. (2014). Impact of bacterial biofilm on the treatment of prosthetic joint infections. J. Antimicrob. Chemother..

[116] Sharma D., Misba L., Khan A.U. (2019). Antibiotics versus biofilm: An emerging battleground in microbial communities. Antimicrob. Resist. Infect. Control..

[117] Khan F., Pham D.T.N., Tabassum N., Oloketuyi S.F., Kim Y.M. (2020). Treatment strategies targeting persister cell formation in bacterial pathogens. Crit. Rev. Microbiol..

[118] Filipović U., Dahmane R.G., Ghannouchi S., Zore A., Bohinc K. (2020). Bacterial adhesion on orthopedic implants. Adv. Colloid. Interface Sci..

[119] Ivanovic B., Trifunovic D., Matic S., Petrovic J., Sacic D., Tadic M. (2019). Prosthetic valve endocarditis—A trouble or a challenge?. J. Cardiol..

[120] Alves C.H., Russi K.L., Rocha N.C., Bastos F., Darrieux M., Parisotto T.M., Girardello R. (2022). Host-microbiome interactions regarding peri-implantitis and dental implant loss. J. Transl. Med..

[121] Zhu Z., Wang Z., Li S., Yuan X. (2019). Antimicrobial strategies for urinary catheters. J. Biomed. Mater. Res. A.

[122] Ng V.Y. (2012). Risk of disease transmission with bone allograft. Orthopedics.

[123] Trobos M., Johansson M.L., Jonhede S., Peters H., Hoffman M., Omar O., Thomsen P., Hultcrantz M. (2018). The clinical outcome and microbiological profile of bone-anchored hearing systems (BAHS) with different abutment topographies: A prospective pilot study. Eur. Arch. Otorhinolaryngol..

[124] Zare M., Ghomi E.R., Venkatraman P.D., Ramakrishna S. (2021). Silicone-based biomaterials for biomedical applications: Antimicrobial strategies and 3D printing technologies. J. Appl. Polym. Sci..

[125] Elbourne A., Chapman J., Gelmi A., Cozzolino D., Crawford R.J., Truong V.K. (2019). Bacterial-nanostructure interactions: The role of cell elasticity and adhesion forces. J. Colloid. Interface Sci..

[126] Wang C., Hou J., van der Mei H.C., Busscher H.J., Ren Y. (2019). Emergent properties in *Streptococcus mutans* biofilms are controlled through adhesion force sensing by initial colonizers. mBio.

[127] Viljoen A., Mignolet J., Viela F., Mathelié-Guinlet M., Dufrêne Y.F. (2020). How microbes use force to control adhesion. J. Bacteriol..

[128] Sousa V., Mardas N., Spratt D., Hassan I.A., Walters N.J., Beltrán V., Donos N. (2022). The effect of microcosm biofilm decontamination on surface topography, chemistry, and biocompatibility dynamics of implant titanium surfaces. Int. J. Mol. Sci..

[129] Folliero V., Franci G., Dell’Annunziata F., Giugliano R., Foglia F., Sperlongano R., De Filippis A., Finamore E., Galdiero M. (2021). Evaluation of antibiotic resistance and biofilm production among clinical strain isolated from medical devices. Int. J. Microbiol..

[130] Tran P.A., Hocking D.M., O’Connor A.J. (2015). In situ formation of antimicrobial silver nanoparticles and the impregnation of hydrophobic polycaprolactone matrix for antimicrobial medical device applications. Mater. Sci. Eng. C Mater. Biol. Appl..

[131] Haidari H., Kopecki Z., Bright R., Cowin A.J., Garg S., Goswami N., Vasilev K. (2020). Ultrasmall AgNP-impregnated biocompatible hydrogel with highly effective biofilm elimination properties. ACS Appl. Mater. Interfaces.

[132] Ivanova K., Fernandes M.M., Mendoza E., Tzanov T. (2015). Enzyme multilayer coatings inhibit *Pseudomonas aeruginosa* biofilm formation on urinary catheters. Appl. Microbiol. Biotechnol..

[133] Rodríguez López A.L., Lee M.R., Ortiz B.J., Gastfriend B.D., Whitehead R., Lynn D.M., Palecek S.P. (2019). Preventing *S. aureus* biofilm formation on titanium surfaces by the release of antimicrobial β-peptides from polyelectrolyte multilayers. Acta Biomater..

[134] Zou Y., Liu C., Zhang H., Wu Y., Lin Y., Cheng J., Lu K., Li L., Zhang Y., Chen H. (2022). Three lines of defense: A multifunctional coating with anti-adhesion, bacteria-killing and anti-quorum sensing properties for preventing biofilm formation of *Pseudomonas aeruginosa*. Acta Biomater..

[135] Saravanan S., Vimalraj S., Thanikaivelan P., Banudevi S., Manivasagam G. (2019). A review on injectable chitosan/beta glycerophosphate hydrogels for bone tissue regeneration. Int. J. Biol. Macromol..

[136] Robert B., Chenthamara D., Subramaniam S. (2022). Fabrication and biomedical applications of arabinoxylan, pectin, chitosan, soy protein, and silk fibroin hydrogels via laccase-ferulic acid redox chemistry. Int. J. Biol. Macromol..

[137] Jalil S.A., Akram M., Bhat J.A., Hayes J.J., Singh S.C., ElKabbash M., Guo C. (2020). Creating superhydrophobic and antibacterial surfaces on gold by femtosecond laser pulses. Appl. Surf. Sci..

[138] Tripathy A., Kumar A., Sreedharan S., Muralidharan G., Pramanik A., Nandi D., Sen P. (2018). Fabrication of low-cost flexible superhydrophobic antibacterial surface with dual-scale roughness. ACS Biomater. Sci. Eng..

[139] Petrini M., Giuliani A., Di Campli E., Di Lodovico S., Iezzi G., Piattelli A., D’Ercole S. (2020). The bacterial anti-adhesive activity of double-etched titanium (DAE) as a dental implant surface. Int. J. Mol. Sci..

[140] D’Ercole S., Cellini L., Pilato S., Di Lodovico S., Iezzi G., Piattelli A., Petrini M. (2020). Material characterization and *Streptococcus oralis* adhesion on Polyetheretherketone (PEEK) and titanium surfaces used in implantology. J. Mater. Sci. Mater. Med..

[141] Hasan J., Jain S., Padmarajan R., Purighalla S., Sambandamurthy V.K., Chatterjee K. (2018). Multi-scale surface topography to minimize adherence and viability of nosocomial drug-resistant bacteria. Mater. Des..

[142] May R.M., Hoffman M.G., Sogo M.J., Parker A.E., O’Toole G.A., Brennan A.B., Reddy S.T. (2014). Micro-patterned surfaces reduce bacterial colonization and biofilm formation in vitro: Potential for enhancing endotracheal tube designs. Clin. Transl. Med..

[143] Xu B., Wei Q., Mettetal M.R., Han J., Rau L., Tie J., May R.M., Pathe E.T., Reddy S.T., Sullivan L. (2017). Surface micropattern reduces colonization and medical device-associated infections. J. Med. Microbiol..

[144] May R.M., Magin C.M., Mann E.E., Drinker M.C., Fraser J.C., Siedlecki C.A., Brennan A.B., Reddy S.T. (2015). An engineered micropattern to reduce bacterial colonization, platelet adhesion and fibrin sheath formation for improved biocompatibility of central venous catheters. Clin. Transl. Med..

[145] Rostami S., Puza F., Ucak M., Ozgur E., Gul O., Ercan U.K., Garipcan B. (2021). Bifunctional sharkskin mimicked chitosan/graphene oxide membranes: Reduced biofilm formation and improved cytocompatibility. Appl. Surf. Sci..

[146] He M.W.Q., Zhao W. (2017). A self-defensive bilayer hydrogel coating with bacteria triggered switching from cell adhesion to antibacterial adhesion. Polym. Chem..

[147] Park J.S., Ryu E.J., Li L., Choi B.K., Kim B.M. (2017). New bicyclic brominated furanones as potent autoinducer-2 quorum-sensing inhibitors against bacterial biofilm formation. Eur. J. Med. Chem..

[148] Olivares E., Badel-Berchoux S., Provot C., Prévost G., Bernardi T., Jehl F. (2019). Clinical impact of antibiotics for the treatment of *Pseudomonas aeruginosa* biofilm infections. Front. Microbiol..

[149] Srinivasan R., Santhakumari S., Poonguzhali P., Geetha M., Dyavaiah M., Lin X. (2021). Bacterial biofilm inhibition: A focused rview on recent therapeutic strategies for combating the biofilm mediated infections. Front. Microbiol..

[150] Cloutier M., Mantovani D., Rosei F. (2015). Antibacterial coatings: Challenges, perspectives, and opportunities. Trends Biotechnol..

[151] Olmo A.D., Rubio R., Martinez S., Perez-Alvarez L., Vilela V.J.C. (2020). Antibacterial coatings for improving the performance of biomaterials. Coatings.

[152] Bruna T., Maldonado-Bravo F., Jara P., Caro N. (2021). Silver nanoparticles and their antibacterial applications. Int. J. Mol. Sci..

[153] Romanò C.L., Scarponi S., Gallazzi E., Romanò D., Drago L. (2015). Antibacterial coating of implants in orthopaedics and trauma: A classification proposal in an evolving panorama. J. Orthop. Surg. Res..

[154] Kuehl R., Brunetto P.S., Woischnig A.K., Varisco M., Rajacic Z., Vosbeck J., Terracciano L., Fromm K.M., Khanna N. (2016). Preventing implant-associated infections by silver coating. Antimicrob. Agents Chemother..

[155] Haktaniyan M., Bradley M. (2022). Polymers showing intrinsic antimicrobial activity. Chem. Soc. Rev..

[156] Asma S.T., Imre K., Morar A., Herman V., Acaroz U., Mukhtar H., Arslan-Acaroz D., Shah S.R.A., Gerlach R. (2022). An Overview of Biofilm Formation-Combating Strategies and Mechanisms of Action of Antibiofilm Agents. Life.

[157] Leng C., Hung H.C., Sun S., Wang D., Li Y., Jiang S., Chen Z. (2015). Probing the surface hydration of nonfouling zwitterionic and PEG materials in contact with proteins. ACS Appl. Mater. Interfaces.

[158] Liu C., Faria A.F., Ma J., Elimelech M. (2017). Mitigation of biofilm development on thin-film composite membranes functionalized with zwitterionic polymers and silver nanoparticles. Environ. Sci. Technol..

[159] Costa B., Martínez-de-Tejada G., Gomes P.A.C., Martins M.C.L., Costa F. (2021). Antimicrobial peptides in the battle against orthopedic implant-related infections: A review. Pharmaceutics.

[160] Barbosa M., Costa F., Monteiro C., Duarte F., Martins M.C.L., Gomes P. (2019). Antimicrobial coatings prepared from Dhvar-5-click-grafted chitosan powders. Acta Biomater..

[161] Guo S., Zhu X., Loh X.J. (2017). Controlling cell adhesion using layer-by-layer approaches for biomedical applications. Mater. Sci. Eng. C Mater. Biol. Appl..

[162] Ahmad D., van den Boogaert I., Miller J., Presswell R., Jouhara H. (2018). Hydrophilic and hydrophobic materials and their applications. Energy Sources Part A Recovery Util. Environ. Eff..

[163] Drago L., Cappelletti L., De Vecchi E., Pignataro L., Torretta S., Mattina R. (2014). Antiadhesive and antibiofilm activity of hyaluronic acid against bacteria responsible for respiratory tract infections. APMIS.

[164] Nemani S.K., Annavarapu R.K., Mohammadian B., Raiyan A., Sojoudi H. (2018). Surface modification: Surface modification of polymers: Methods and applications. Adv. Mater. Interfaces.

[165] Al-Gunaid T.A., Krupa I., Ouederni M., Krishnamoorthy S.K., Popelka A. (2021). Enhancement of adhesion characteristics of low-density polyethylene using atmospheric plasma iitiated-grafting of polyethylene glycol. Polymers.

[166] Sterzenbach T., Helbig R., Hannig C., Hannig M. (2020). Bioadhesion in the oral cavity and approaches for biofilm management by surface modifications. Clin. Oral. Investig..

[167] Nemani S.K., Annavarapu R.K., Mohammadian B., Raiyan A., Heil J., Haque M.A., Abdelaal A., Sojoudi H. (2018). Surface Modification of Polymers: Methods and Applications. Adv. Mater. Interfaces.

[168] Klodzinska S.N., Wan F., Jumaa H., Sternberg C., Rades T., Nielsen H.M. (2019). Utilizing nanoparticles for improving anti-biofilm effects of azithromycin: A head-to-head comparison of modified hyaluronic acid nanogels and coated poly (lactic-co-glycolic acid) nanoparticles. J. Colloid. Interface Sci..

[169] Yadav J., Kumari R.M., Verma V., Nimesh S. (2021). Recent development in therapeutic strategies targeting *Pseudomonas aeruginosa* biofilms—A review. Mater. Today Proc..

[170] Encinas N., Pantoja M., Abenojar J., Martínez M.A. (2010). Control of wettability of polymers by surface roughness modification. J. Adhes. Sci. Technol..

[171] Epperlein N., Menzel F., Schwibbert K., Koter R., Bonse J., Sameith J., Krueger J., Toepel J. (2017). Influence of femtosecond laser produced nanostructures on biofilm growth on steel. Appl. Surf. Sci..

[172] Gillett A., Waugh D., Lawrence J., Swainson M., Dixon R. (2016). Laser surface modification for the prevention of biofouling by infection causing *Escherichia coli*. J. Laser Appl..

[173] Zhang Y., Jiang Q., Long M., Han R., Cao K., Zhang S., Feng D., Jia T., Sun Z., Qiu J. (2022). Femtosecond laser-induced periodic structures: Mechanisms, techniques, and applications. Opto-Electron. Sci..

[174] Monteiro D.R., de Souza Batista V.E., Caldeirão A.C.M., Jacinto R.C., Pessan J.P. (2021). Oral prosthetic microbiology: Aspects related to the oral microbiome, surface properties, and strategies for controlling biofilms. Biofouling.

[175] Giner L., Mercadé M., Torrent S., Punset M., Pérez R.A., Delgado L.M., Gil F.J. (2018). Double acid etching treatment of dental implants for enhanced biological properties. J. Appl. Biomater. Funct. Mater..

[176] Najeeb S., Zafar M.S., Khurshid Z., Siddiqui F. (2016). Applications of polyetheretherketone (PEEK) in oral implantology and prosthodontics. J. Prosthodont. Res..

[177] Wang J., Wu Y., Cao Y., Li G., Liao Y. (2020). Influence of surface roughness on contact angle hysteresis and spreading work. Colloid Polym. Sci..

[178] Wu S., Altenried S., Zogg A., Zuber F., Maniura-Weber K., Ren Q. (2018). Role of the surface nanoscale roughness of stainless steel on bacterial adhesion and microcolony formation. ACS Omega.

[179] Kozmos M., Virant P., Rojko F., Abram A., Rudolf R., Raspor P., Zore A., Bohinc K. (2021). Bacterial adhesion of *Streptococcus mutans* to dental material surfaces. Molecules.

[180] Lu A., Gao Y., Jin T., Luo X., Zeng Q., Shang Z. (2020). Effects of surface roughness and texture on the bacterial adhesion on the bearing surface of bio-ceramic joint implants: An in vitro study. Ceram. Int..

[181] Matos G.R.M. (2021). Surface roughness of dental implant and osseointegration. J. Maxillofac. Oral. Surg..

[182] Nazarov D.V., Smirnov V.M., Zemtsova E.G., Yudintceva N.M., Shevtsov M.A., Valiev R.Z. (2018). Enhanced osseointegrative properties of ultra-fine-grained titanium implants modified by chemical etching and atomic layer deposition. ACS Biomater. Sci. Eng..

[183] Wardhono E.Y., Pinem M.P., Susilo S., Siom B.J., Sudrajad A., Pramono A., Meliana Y., Guénin E. (2022). Modification of physio-mechanical properties of chitosan-based films via physical treatment approach. Polymers.

[184] Kumar A., Seenivasan M.K., Inbarajan A. (2021). A literature review on biofilm formation on silicone and poymethyl methacrylate used for maxillofacial prostheses. Cureus.

[185] Desrousseaux C., Sautou V., Descamps S., Traoré O. (2013). Modification of the surfaces of medical devices to prevent microbial adhesion and biofilm formation. J. Hosp. Infect..

[186] Renner L.D., Weibel D.B. (2011). Physicochemical regulation of biofilm formation. MRS Bull..

[187] Xiong F.Z., Zhao X.X., Liao Y.H. (2018). Effect of material surface characteristics on biofilm formation and its application. Microbiol. China.

[188] Freitas S.C., Correa-Uribe A., Martins M.C.L., Pelaez-Vargas A. (2018). Self-assembled monolayers for dental implants. Int. J. Dent..

[189] Somasundaram S. (2018). Silane coatings of metallic biomaterials for biomedical implants: A preliminary review. J. Biomed. Mater. Res. B Appl. Biomater..

[190] Nicosia C., Huskens J. (2014). Reactive self-assembled monolayers: From surface functionalization to gradient formation. Mater. Horiz..

[191] Chandrasekaran M., Kim K.D., Chun S.C. (2020). Antibacterial Activity of Chitosan Nanoparticles: A Review. Processes.

[192] Tan W.-S., Law J.W.-F., Law L.N.-S., Letchumanan V., Chan K.-G. (2020). Insights into quorum sensing (QS): QS-regulated biofilm and inhibitors. Prog. Microbes Mol. Biol..

[193] Sionov R.V., Steinberg D. (2022). Targeting the holy triangle of quorum sensing, biofilm formation, and antibiotic resistance in pathogenic bacteria. Microorganisms.

[194] Vashistha A., Sharma N., Nanaji Y., Kumar D., Singh G., Barnwal R.P., Yadav A.K. (2023). Quorum sensing inhibitors as Therapeutics: Bacterial biofilm inhibition. Bioorganic Chem..

[195] Zhou L., Zhang Y., Ge Y., Zhu X., Pan J. (2020). Regulatory mechanisms and promising applications of quorum sensing-inhibiting agents in control of bacterial biofilm Formation. Front. Microbiol..

[196] Zhang Q., Li S., Hachicha M., Boukraa M., Soulère L., Efrit M.L., Queneau Y. (2021). Heterocyclic chemistry applied to the design of N-Acyl homoserine lactone analogues as bacterial quorum sensing signals mimics. Molecules.

[197] Kolenbrander P.E., Palmer R.J., Periasamy S., Jakubovics N.S. (2010). Oral multispecies biofilm development and the key role of cell-cell distance. Nat. Rev. Microbiol..

